# A Multi-Mineral Intervention to Modulate Colonic Mucosal Protein Profile: Results from a 90-Day Trial in Human Subjects

**DOI:** 10.3390/nu13030939

**Published:** 2021-03-14

**Authors:** Muhammad N. Aslam, Shannon D. McClintock, Mohamed Ali H. Jawad-Makki, Karsten Knuver, Haris M. Ahmad, Venkatesha Basrur, Ingrid L. Bergin, Suzanna M. Zick, Ananda Sen, D. Kim Turgeon, James Varani

**Affiliations:** 1 Department of Pathology, The University of Michigan Medical School, Ann Arbor, MI 48109, USA; mcclint@umich.edu (S.D.M.); mjmakki@umich.edu (M.A.H.J.-M.); kknuver@med.umich.edu (K.K.); ahmadha@umich.edu (H.M.A.); vbasrur@med.umich.edu (V.B.); varani@med.umich.edu (J.V.); 2 The Unit for Laboratory Animal Medicine, The University of Michigan Medical School, Ann Arbor, MI 48109, USA; ingridbe@med.umich.edu; 3 Department of Family Medicine, The University of Michigan Medical School, Ann Arbor, MI 48109, USA; szick@med.umich.edu (S.M.Z.); anandas@med.umich.edu (A.S.); 4 Department of Nutritional Science, The University of Michigan School of Public Health, Ann Arbor, MI 48109, USA; 5 Department of Biostatistics, The University of Michigan Medical School, Ann Arbor, MI 48109, USA; 6 Department of Internal Medicine (Division of Gastroenterology), The University of Michigan Medical School, Ann Arbor, MI 48109, USA; kturgeon@med.umich.edu

**Keywords:** Aquamin^®^, biomarkers, calcium, colon cancer chemoprevention, minerals, proteomic analysis, trace elements

## Abstract

The overall goal of this study was to determine whether Aquamin^®^, a calcium-, magnesium-, trace element-rich, red algae-derived natural product, would alter the expression of proteins involved in growth-regulation and differentiation in colon. Thirty healthy human subjects (at risk for colorectal cancer) were enrolled in a three-arm, 90-day interventional trial. Aquamin^®^ was compared to calcium alone and placebo. Before and after the interventional period, colonic biopsies were obtained. Biopsies were evaluated by immunohistology for expression of Ki67 (proliferation marker) and for CK20 and p21 (differentiation markers). Tandem mass tag-mass spectrometry-based detection was used to assess levels of multiple proteins. As compared to placebo or calcium, Aquamin^®^ reduced the level of Ki67 expression and slightly increased CK20 expression. Increased p21 expression was observed with both calcium and Aquamin^®^. In proteomic screen, Aquamin^®^ treatment resulted in many more proteins being upregulated (including pro-apoptotic, cytokeratins, cell–cell adhesion molecules, and components of the basement membrane) or downregulated (proliferation and nucleic acid metabolism) than placebo. Calcium alone also altered the expression of many of the same proteins but not to the same extent as Aquamin^®^. We conclude that daily Aquamin^®^ ingestion alters protein expression profile in the colon that could be beneficial to colonic health.

## 1. Introduction

It is well-accepted that an adequate intake of dietary calcium is important for the prevention of several long-latency diseases [1,2,3,4,5] including colorectal cancer [6,7,8,9,10,11]. While calcium (as a single mineral) has been widely studied, it is now becoming recognized that other minerals along with calcium can provide benefit not obtainable with calcium alone [12,13,14]. Some of these (e.g., chromium, copper, iron, magnesium, manganese, molybdenum, potassium, selenium, sodium, and zinc) have recommended dietary intake levels [15,16,17,18,19], but others are utilized in such small amounts that intake levels have not been defined. Past studies in our laboratory have made use of a multi-mineral natural product (Aquamin^®^) as part of a strategy for assessing the role of minerals in the prevention of long-latency diseases. Aquamin^®^ is derived from the mineralized remains of red marine algae and consists of calcium and magnesium along with detectable levels of 72 additional trace elements [20,21,22,23,24,25,26,27].

In regard to colon cancer, specifically, calcium alone has been tested for antitumor efficacy in mouse models [28] and in vitro studies where it modulates colon epithelial cell proliferation and differentiation [29]. However, results from interventional (polyp prevention) trials in humans are mixed, with some trials having demonstrated a decrease in the incidence [30,31], while others showing no beneficial effect [32,33]. This suggests that effective colon cancer chemoprevention may not be straightforward. It may require beginning intervention early in life [34], and may also depend on the inclusion of additional minerals or trace elements in addition to calcium alone. To this end, we have demonstrated that Aquamin^®^ reduces proliferation and induces differentiation of human colon carcinoma cells in monolayer culture [20,21] and in organoid culture [22,23,24,25] more effectively than calcium alone. Along with these in vitro studies, two long-term (15–18 month) studies in mice demonstrated the improved efficacy of Aquamin^®^ relative to that of calcium alone for inhibition of colon polyp formation and reduction of inflammation [26,27], when a multi-mineral intervention was begun early and continued over the lifespan of the animals.

While these findings provide evidence for efficacy of the multi-mineral approach to disease prevention, they do not indicate whether a multi-mineral supplement such as Aquamin^®^ can be used in humans or whether the same beneficial activities observed in preclinical models will be seen in humans. To begin addressing this issue, we carried out a 90-day, United States Food and Drug Administration (FDA)-approved trial, comparing daily intervention with Aquamin^®^ to calcium alone and placebo in healthy human subjects but at increased risk for colorectal cancer (CRC). While the primary purpose of the study was to assess safety and tolerability, we also evaluated microbial community structure and assessed bile acid, short-chain fatty acid (SCFA), and eicosanoid profiles at endpoint (after 90-days of intervention) in comparison to baseline values in subjects from all three groups. Those data have been published recently [35]. As part of the same study, replicate colonic biopsies were obtained from each subject prior to intervention and at the end of the 90-day treatment period. One set of biopsies was evaluated morphometrically for histological changes (crypt length) and by quantitative immunohistology for expression of Ki67 as a proliferation marker, and for expression of CK20 and p21 as indicators of differentiation. These biomarkers of risk for colorectal cancer have been used in past colon polyp prevention biomarker studies [19,36,37,38]. Additional biopsies from each cohort of individuals were subjected to proteomic profile evaluation via tandem mass tag (TMT) labeled mass spectrometry. The results of the histological/immunohistological and proteomic studies are described in the current report. Findings from the interventional trial are compared to recent findings obtained using similar approaches in colon organoid culture [22,23,24].

## 2. Materials and Methods

### 2.1. Interventions

Three study interventions were used, one experimental (Aquamin^®^) and two comparators (calcium carbonate and maltodextrin). Calcium carbonate was used as an active comparator and maltodextrin was used as a placebo. Aquamin^®^ is a natural product obtained from the skeletal remains of red marine algae of the *Lithothamnion* genus [39]. Aquamin^®^ is approximately 30% calcium (by weight), contains calcium and magnesium in an approximately 14:1 molar ratio and has detectable levels of 72 additional trace minerals including trace elements from the lanthanide family (essentially all of the minerals accumulated by the algae from seawater). Aquamin^®^ is sold as a food supplement (Generally Recognized as Safe - GRAS 000028) (Marigot Ltd., Cork, Ireland) and is used in various products for human consumption in Europe, Asia, Australia, and North America. A single batch of Aquamin-Food Grade^®^ was used for this study. The mineral composition was established via an independent laboratory (Advanced Laboratories; Salt Lake City, UT, USA) using inductively coupled plasma-optical emission spectrometry (ICP-OES). A complete list of elements detected in this batch of Aquamin^®^ and their relative amounts can be found in our recent report [35] and in Table S1. Table S1 provides estimated amounts of these individual minerals and trace elements as present in a daily dose of Aquamin^®^. Daily intake of Aquamin^®^ or calcium alone was designed to provide 800 mg of calcium per day. Aquamin^®^ has been used in past clinical studies in human subjects without any reported serious safety or tolerability issues [35,40,41,42].

### 2.2. Trial Design

The design of this 90-days interventional study was described in detail in our earlier publication [35]. Briefly, this was a placebo-controlled, double-blinded, parallel assigned trial in which thirty subjects were included. Thirty subjects were randomized (by masking participants and investigators) into three arms, allocating 10 subjects per arm. These subjects were 18 to 80 years aged, male or female (non-pregnant/non-lactating). Subjects were in general good health but with “an increased risk for colon cancer” based on a personal history of (i) colorectal polyp, (ii) early-stage (stage I or II) colon cancer (surgically removed without administration of adjuvant therapy), (iii) stage III colon cancer treated with surgery more than 5 years ago, or (iv) having a first-degree blood relative diagnosed with colorectal cancer under the age of 60 years. Exclusion criteria included history of kidney disease or kidney stones, Crohn’s disease or ulcerative colitis, gastrointestinal hemorrhagic disorders, or coagulopathy, hereditary non-polyposis coli or familial adenomatous polyposis.

Baseline serum calcium levels were evaluated by using NIH Diet History Questionnaire II (DHQ II), a food frequency questionnaire that also inquires about dietary supplement usage in the past twelve months at the screening visit. Subjects were also asked about any current use of dietary supplements, antibiotics, steroids, and nonsteroidal anti-inflammatory drugs. Subjects were required to stop ingesting calcium and/or vitamin D containing supplements, steroids, and nonsteroidal anti-inflammatory drugs two-weeks before starting and during the study participation.

This FDA-approved interventional trial was conducted at Michigan Medicine under an Investigational New Drug (IND) #118194. The Institutional Review Board at the University of Michigan Medical School (IRBMED) provided the required oversight. The study was listed on Clinicaltrials.gov (study identifier NCT02647671). The subjects were recruited through the Michigan Medicine web portal (umhealthresearch.org) and by posting flyers in the hospital. All subjects provided written informed consent before participation. Subjects were not charged for study-related procedures and were appropriately compensated for their participation. This phase I interventional trial involving human subjects was conducted in accordance with recognized ethical guidelines, for example, the Declaration of Helsinki, International Ethical Guidelines for Biomedical Research Involving Human Subjects (CIOMS), the Belmont Report and the U.S. Common Rule.

Subjects underwent a flexible sigmoidoscopy procedure at baseline. No bowel cleansing was performed prior to the procedure. Twelve 2.5 mm colonic mucosal biopsies were obtained using endoscopic pinch biopsy from normal-looking colon lining along with two stool specimens from within the sigmoid colon (20 cm above the anus). After baseline sigmoidoscopy, subjects were randomized to one of three groups. Ten subjects were given Aquamin^®^ capsules for 90 days, providing 800 mg of calcium per day. Ten subjects received 800 mg of calcium carbonate daily and ten subjects received maltodextrin as placebo. At the end of the 90-day intervention period (90 ± 5 days), subjects again underwent unprepped flexible sigmoidoscopy. Eight colonic mucosal biopsies along with two stool specimens were collected. Pre- and post-intervention tissue biopsies and stool samples were snap-frozen in liquid nitrogen and saved at −80 °C. A set of colon mucosal biopsies were fixed in 10% buffered formalin. A venous blood sample was drawn for serum liver function and injury markers at both visits. For each of the two time-points, one biopsy and one stool specimen from each participant was utilized for microbiome analysis and one biopsy and one stool specimen was utilized for metabolomic profiling. The remaining tissue samples were used for histological, immunohistochemical, and proteomic analyses. Microbiome/metabolomic data from this trial have been published along with safety and tolerability findings [35]. This report presents findings from histology/immunohistology and initial proteomic analysis-direct effects of Aquamin^®^ on the colonic epithelium.

During the participation phase, study coordinators remotely contacted subjects monthly to assess adherence to the study protocol and to identify any adverse events. Study compliance was evaluated by counting unused capsules and capsule log entries returned at the end of the study. Compliance (by capsule count) was estimated to be 96% across the three groups. No subject withdrew once enrolled in the study and started an intervention. There was no reportable serious adverse event that occurred with Aquamin^®^. All adverse events were minor in nature and did not disqualify any subject from completing study participation. The details can be found in a recent report [35].

### 2.3. Histology and Quantitative Immunohistochemistry

Baseline and post-intervention colon mucosal biopsies were fixed in 10% buffered formalin and embedded in paraffin. Paraffin-embedded biopsies were sectioned and stained with hematoxylin and eosin (H&E) and examined by light microscopy for tissue orientation and full-length crypt evaluation.

For immunohistochemistry (IHC), paraffin-embedded tissue was sectioned and assessed for markers for proliferation (Ki67) and differentiation (Cytokeratin20 [CK20] and p21 [or WAF1]). Table S2 provides a list of antibodies used, their source, and relevant characteristics. These antibodies have been tested and validated for the formalin-fixed, paraffin-embedded tissue. Briefly, colonic samples were sectioned at 5 μm thickness, deparaffinized, rehydrated in graded ethanol solutions, and subjected to heat-induced epitope retrieval with high pH or low pH FLEX TRS Retrieval buffer (Agilent Technologies, 154 #K8004; Santa Clara, CA, United States) for 20 min. After peroxidase blocking, antibodies were applied at appropriate dilutions at room temperature for 30 or 60 min depending on the manufacturer’s recommendation. The FLEX HRP EnVision System (Agilent Technologies) was used for detection with a 10-min DAB chromagen application. Control tissue was used to test for negative and positive staining.

### 2.4. Quantitative Morphometry

Colonic sections of stained and immunostained tissue on glass slides were digitally scanned (at 40×) using the Aperio AT2 brightfield whole slide scanner (Leica Biosystems) at a resolution of 0.5 µm per pixel with 20× objective for quantitative image analysis. The scanned images were housed on a secure server and remotely accessed using Leica Aperio eSlide Manager (v12.4.3.5008), a digital pathology management software. A slide-viewing software by Aperio ImageScope (v12.4.3.5008) and associated image analysis tools were used to examine and quantitate these scanned histological sections. The quantitation was performed blindly by masking the subject IDs.

For crypt length measurement, digitized H&E stained sections were analyzed to evaluate full-length crypts by measuring the height of each crypt from the base of the crypt to the mucosal surface. Crypt lengths were averaged for each slide, grouped and compared with the other groups.

Brightfield Immunohistochemistry Image Analysis tools (Leica) were used to quantify and interpret biomarker expression of the three immunostains used in this study. Crypts were selected and lamina propria was excluded by using the annotation tool, on the entire length of the tissue section. Markup images were checked for the accuracy of the quantitation. Aperio Nuclear Algorithm (v9) was used for Ki67 and p21 expression quantification. This algorithm measures intensity of the nuclear staining in individual cells and separates those into very intense to no nuclear staining (3+, 2+, 1+, and 0, respectively). Percent positive nuclei within the crypts were used here for comparison. Nuclei (of cells) in the lamina propria and submucosa were not included. To quantify CK20 expression, the Aperio Positive Pixel Count Algorithm (v9) was used. It quantifies the number and intensity of pixels of a specific stain in a digitized image. Positivity was calculated with numbers of positive pixels against total pixels present within the colonic crypts and mucosal surface.

### 2.5. Proteomic Assessment

Proteomic experiments were performed at the Proteomics Resource Facility (PRF) in the Department of Pathology at the University of Michigan, employing mass spectrometry (MS)-based tandem mass tag (TMT) analysis (ThermoFisher Scientific) for the relative quantification of proteins. Individual colon biopsies were weighed (the weights were ranged from 5.5 mg to 12 mg) and exposed to radioimmunoprecipitation assay (RIPA) lysis and extraction buffer (Thermo Scientific cat#89901) at 25 µL per mg for protein. After that, biopsies were manually homogenized employing 1.5 mL tube sample pestles (RPI item#199228) and then freeze fractured for 1 cycle. Samples were centrifuged at 20,000× *g* for 10 min to remove insoluble material. Supernatants were removed to a clean tube and then protein concentrations were determined using a BCA assay kit (Pierce cat#23227). Following this, individual samples were “pooled” in groups of five (i.e., 5 placebo, 5 calcium, and 5 Aquamin^®^ specimens, before and after intervention), allowing us to evaluate all 30 samples in two TMTsixplex runs. Each pool was comprised of 8 micrograms per sample, 5 samples per pool for a total of 40 µg. The volume was adjusted such that each pool was 40 µg of pooled protein in 40 µL of total volume. Briefly, forty micrograms of protein from each pool were separately digested with trypsin and individually labeled with one of the 6 isobaric mass tags following the manufacturer’s protocol using TMTsixplex kit (ThermoFisher cat# 90061) for each MS analysis. After labeling, equal amounts of the peptide from each pool were mixed together. In order to achieve in-depth characterization of the proteome, the labeled peptides were fractionated using 2D-LC (basic pH reverse-phase separation followed by acidic pH reverse-phase) and analyzed on a high-resolution, Tribrid mass spectrometer (Orbitrap Fusion Tribrid, ThermoFisher Scientific) using conditions optimized at the PRF. MultiNotch MS3 approach [43] was employed to obtain accurate quantitation of the identified proteins/peptides. Data analysis was performed using Proteome Discoverer (v2.4, ThermoFisher). MS2 spectra were searched against UniProt human protein database (downloaded on 06/20/2019; 20,353 reviewed entries) using the following search parameters: MS1 and MS2 tolerance were set to 10 ppm and 0.6 Da, respectively; carbamidomethylation of cysteines (57.02146 Da) and TMT labeling of lysine and N-termini of peptides (229.16293 Da) were considered static modifications; oxidation of methionine (15.9949 Da) and deamidation of asparagine and glutamine (0.98401 Da) were considered variables. Identified proteins and peptides were filtered to retain only those that passed ≤2% false-discovery rate (FDR) threshold of detection. Quantitation was performed using high-quality MS3 spectra (average signal-to-noise ratio of 6 and 75% isolation interference). Protein names were retrieved using Uniprot.org, and Reactome v74 (reactome.org) was used for initial pathway enrichment analysis for species “Homo sapiens”. STRING database-v11 (string-db.org) was used to detect protein–protein interactions and additional enrichment analyses provide information related to cellular components, molecular functions, and biological processes by Gene Ontology (GO) annotation. It also offered Reactome and Kyoto Encyclopedia of Genes and Genomes (KEGG) databases to curate pathways. Only proteins with *a* ≤ 2% FDR confidence were included in the analyses. The differential protein expression profiles were established by calculating the abundance ratios of normalized abundances of post-intervention samples to pre-intervention samples. For the final comparison, the pre-intervention samples from all 30 subjects and the post-intervention samples from the placebo group were combined to serve as a control. Final abundance ratios of post-intervention samples were calculated and compared to this control. It should be noted that, when only the respective pre-treatment values for each cohort were used as control or when the pre-treatment values from the three cohorts were combined (without the post-treatment placebo) were used to assess proteomic expression, the findings were similar.

The initial analysis involved an unbiased proteome-wide screen of all proteins modified by Aquamin^®^ or by calcium alone in relation to placebo. A follow-up analysis was targeted toward proliferation, differentiation, and barrier-related cell adhesion proteins. Mass spectrometry-based proteomics data were deposited to the ProteomeXchange Consortium via the PRIDE partner repository with the dataset identifier PXD024445.

### 2.6. Data Analysis and Statistical Evaluation

Pre- post-intervention ordinates were obtained for each discrete morphological and immunohistochemical feature. Means and standard deviations were generated, and data were analyzed by ANOVA followed by the two-stage linear step-up procedure of Benjamini, Krieger and Yekutieli for multiple comparison (by controlling FDR). For correlation between two markers, Pearson correlation coefficient was computed at 95% confidence interval with two-tailed *p*-value < 0.05. GraphPad Prism v8.3 was used for these analyses. Proteomic data were generated in two separate runs of six pooled samples per run. Data from the two runs were combined. Results from subjects treated with Aquamin^®^ or calcium alone were compared to results obtained with the placebo. Pathways enrichment data reflect Reactome-generated *p*-values based on the number of entities identified in a given pathway compared to total proteins involved in that pathway. For STRING enrichment analysis, the whole genome statistical background was assumed and FDR stringency was high (≤1%). Data were considered significant at *p* and *q* <0.05.

Due to small group size, analyses were not adjusted to any baseline sociodemographic or clinical features or dietary calcium or mineral intake.

## 3. Results

### 3.1. Histological and Immunohistological Findings: Comparison of Aquamin^®^ with Calcium Alone and Placebo

**Figure 1:**
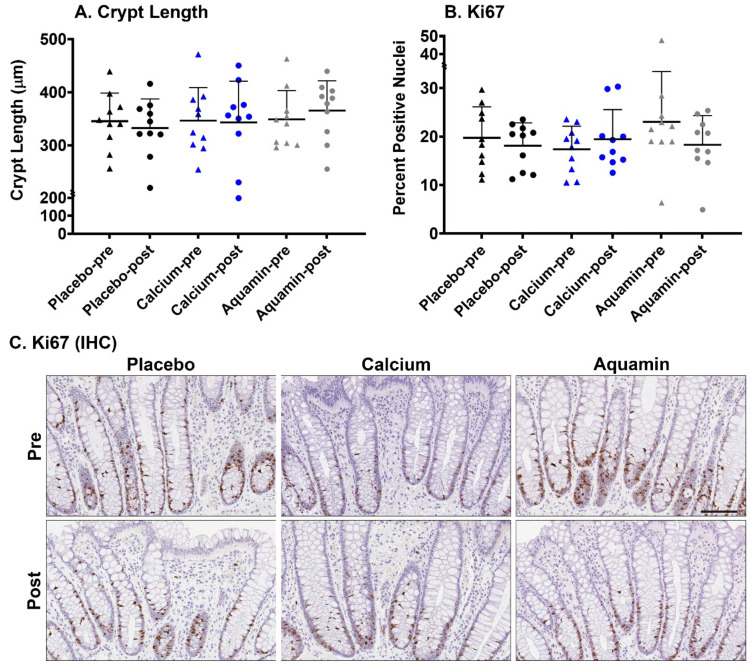
Histological features of the colonic mucosa and proliferation expression. (**A**): Crypt Length. Values represent means and standard deviations based on measurement of crypt length in individual crypts in each colon biopsy (P:70 and 85, CA:2 and 71, AQ:74 and 96–number of pre and post crypts, respectively) per treatment group (10 subjects per group before and after treatment). (**B**): Ki67 expression quantitation. Percentage of Ki67-positive nuclei is presented in each group. Values represent means and standard deviations based on evaluation of individual crypts in each colon biopsy (P:106 and 81, CA:63 and 65, AQ:102 and 82–number of pre and post crypts respectively) per treatment group (10 subjects per group before and after 90-day intervention). Each dot represents an individual subject (**A**,**B**). (**C**): Histological appearance and Ki67-stained histological images of the colonic mucosa from a representative subject in each treatment group before and after the 90-day intervention. Scale bar = 100 µm.

In the first series of studies, colonic biopsies were examined at the light microscopic level for overall crypt appearance and any histological abnormality and were assessed morphometrically for crypt length. No histological abnormality or lesion was found in any of the colon sections. Crypt length was chosen since previous studies have demonstrated little change in this parameter with calcium intervention [38]. Crypt length measurement data from all 30 subjects (10 in each group) are presented in Figure 1A and Figure S1A. As can be seen from Figure 1A, there were no apparent effects of the interventions on crypt length as compared to the placebo group.

Results (quantitative data) of immunostaining studies with Ki67 are shown in Figure 1B. Images of representative sections of pre- and post-treatment biopsies from one subject in each cohort are presented in Figure 1C. Values from the placebo group and calcium group did not differ significantly between pre-treatment and post-treatment. In the Aquamin^®^-treated cohort, however, there was 20% reduction in the average post-treatment value compared to average pre-treatment value. As expected, virtually all of the Ki67 staining was in cells occupying the lower third of the crypt (proliferative zone). The complete pre-post change data (from each individual) are presented in Figure S1B.

**Figure 2:**
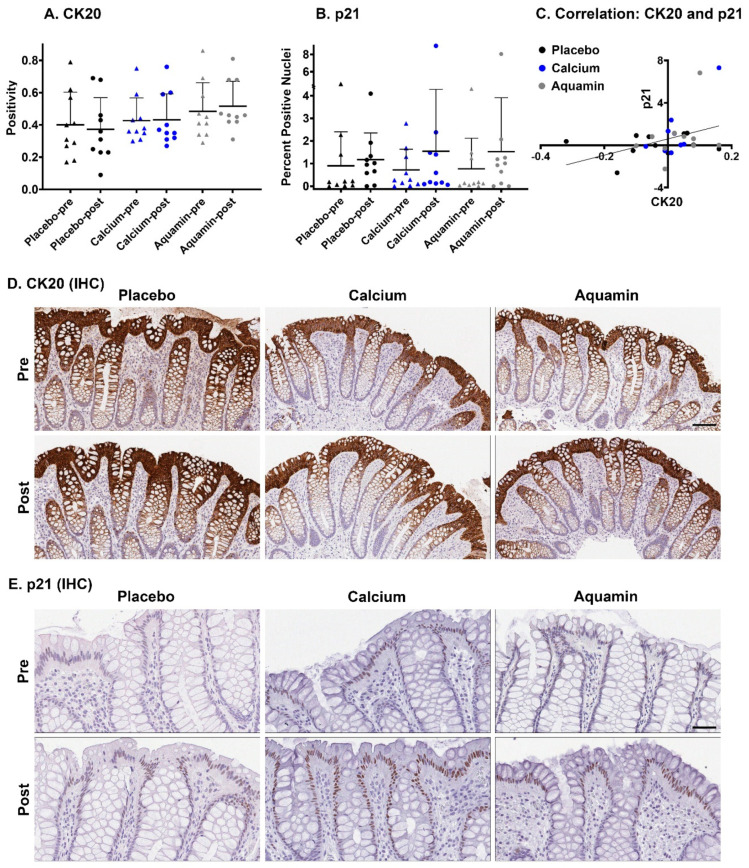
Differentiation panel expression. (**A**): CK20 expression quantitation. CK20 expression is presented by CK20 stain positivity. Values represent means and standard deviations based on evaluation of individual crypts in each colon biopsy (P:106 and 81, CA:63 and 65, AQ:102 and 82–number of pre and post crypts respectively) and luminal surface per treatment group (10 subjects per group before and after treatment for 90 days). (**B**): p21 expression quantitation. Percentage of strong positive (2+ and 3+) nuclei is used to present p21 expression. Values represent means and standard deviations based on evaluation of individual crypts in each colon biopsy (P:106 and 78, CA:59 and 63, AQ:103 and 65–number of pre and post crypts respectively) and luminal epithelial cells per treatment group (10 subjects per group before and after treatment for 90 days). (**C**): Correlation of CK20 and p21 expressions in all 30 subjects. *r* = 0.3819; *p* (two-tailed) = 0.0373. Each dot represents an individual subject (**A**–**C**). (**D**): CK20-stained histological images of colonic mucosa from a representative subject of each treatment group before and after the 90-day intervention. Scale bar = 100 µm. (**E**): p21-stained histological images of colonic mucosa from a representative subject of each treatment group before and after the 90-day intervention. Scale bar = 50 µm.

Figure 2 presents immunostaining results with CK20 and p21. With CK20, no change in expression was observed with placebo or calcium, however, 7% increase in CK20 expression was observed with Aquamin^®^ (Figure 2A). In both pre- and post-intervention specimens, there was strong brown staining visible on the luminal mucosal surface and the top one-third of the full-length crypts (Figure 2D). With p21 quantitation (Figure 2B), there was an increased expression with both calcium alone (average increase of 117%) and Aquamin^®^ (average increase of 99%) in post-intervention biopsies compared to baseline expression.

Positive nuclear staining with p21 was visible in cells on the mucosal surface and the top half of colonic crypts (Figure 2E). Regarding p21 expression, the response to both interventions was not uniform and subject-to-subject variability was evident. The trend toward increased p21 expression in response to calcium is consistent with previous findings [36,37]. The pre-post change data for CK20 and p21 from each individual are presented in Figure S1C,D, respectively.

As part of the immunohistochemical analyses, we searched for correlations between marker expression patterns. Neither CK20 nor p21 expression demonstrated a (positive or negative) correlation with Ki67 expression but the two differentiation markers were positively correlated (*p* = 0.0373) with each other (Figure 2C).

### 3.2. Proteomic Findings: Unbiased Comparison of Aquamin^®^ with Calcium Alone and Placebo

**Figure 3:**
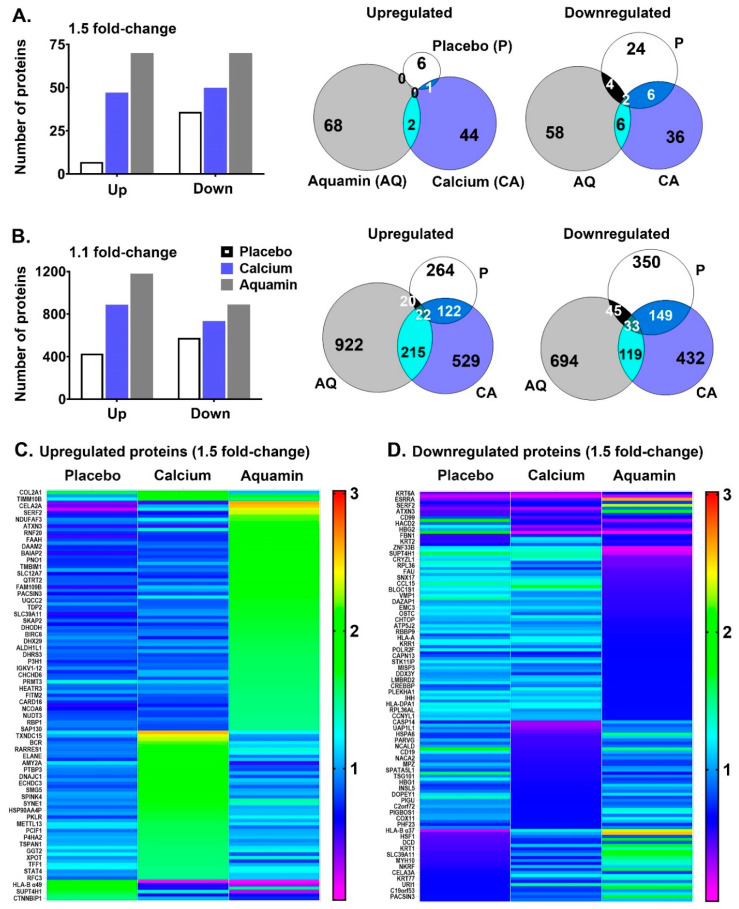
Proteomic expression of colon mucosal biopsies and response to interventions. (**A**): Upregulated and downregulated proteins in each cohort at 1.5-fold. Each bar represents the number of proteins up-regulated (1.5-fold; ≤1% FDR) or down-regulated (0.67-fold; ≤1% FDR) over the 90-day course of treatment with each of the three interventions. On the right, Venn diagrams show the overlap of these upregulated and downregulated moieties to provide common proteins among 3 groups or between 2 groups or unique to an intervention. The list of these upregulated proteins is presented in Table S3. While downregulated proteins are presented in Table S4. (**B**): Upregulated and downregulated proteins in each cohort at 1.1-fold. Each bar represents the number of proteins up-regulated (1.1-fold; ≤1% FDR) or down-regulated (0.9-fold; ≤1% FDR) over the 90-day course of treatment with each of the three interventions. On the right, Venn diagrams show the overlap of these upregulated and downregulated moieties to provide common proteins among 3 groups or between 2 groups or unique to an intervention. (**C**): Differential proteomic expression of upregulated proteins (of all interventions at 1.5-fold; ≤1% FDR) is presented in a heatmap. These proteins are listed in Table S3. (**D**): Differential proteomic expression of downregulated proteins (of all interventions at 1.5-fold; ≤1% FDR) is presented in a heatmap. These proteins are listed in Table S4.

Each of the three interventions was assessed for effects on protein expression profile as described in the Materials and Methods section. A total of 5395 distinct proteins appeared in the mass spectrometry (MS)-based TMT analysis with FDR confidence of ≤1%. An overview of the initial unbiased proteomic screen is presented in Figure 3. The pooled data from all three pre-treatment groups and the post-placebo treatment group were used as a control to give the most comprehensive view of the protein-expression changes in response to each intervention. The data presented in Figure 3A were calculated at 1.5-fold change. The bar graphs on the top left represent the total number of altered proteins at 1.5-fold with ≤1% FDR for each of the three interventions. On the right, two Venn diagrams are presented to show the overlap in altered proteomic expression among the three interventions. It is evident from these data that most of the protein alterations (either up- or downregulated) occurred in response to Aquamin^®^ or calcium alone; there was minimal change in the placebo control group. Similarly, when the stringency was lenient (1.1-fold with ≤1%FDR), the overall trend was the same; that is, most of the alteration was seen with Aquamin^®^ or calcium (Figure 3B). Aquamin^®^ still showed the highest change in quantitative proteomic expression. A heatmap was used to present the differences in proteomic expression among three interventions at 1.5-fold change, upregulated in Figure 3C and downregulated in Figure 3D. Our conclusion from the unbiased analysis is that the 90-day period of treatment with either Aquamin^®^ or calcium alone was sufficient to detect an effect on the protein expression pattern in the colonic mucosa of healthy adult volunteers that was not observed with placebo.

As part of the analysis, we identified individual proteins responsible for altered expression at 1.5-fold change. The individual proteins (assessed by unbiased screen) are presented in Tables S3 (upregulated proteins) and S4 (downregulated proteins). The proteins presented in Tables S3 and S4 were sorted based on the overlap among all three interventions or between two interventions or unique to each intervention. As a next step, we used STRING enriched analysis to highlight any protein–protein interactions among proteins presented in Tables S3 and S4. Some clusters of proteins were evident by having multiple interactions among the participating proteins (Figure S2).

Similarly, Table S5 lists all the STRING-enriched GO biological processes, molecular functions, and cellular components significantly (with *q* < 0.05) altered with the same set of unbiased proteins (1.5-fold; ≤1% FDR). In the final step, significantly altered pathways (with *q* < 0.05) were identified (by Reactome and KEGG curated in STRING) using unbiased proteins altered at 1.5-fold (shown in Figure S3). The top pathways involved by the altered proteins were following; keratinization, cornified envelope formation, hemidesmosome assembly, and collagen-related pathways were upregulated and pathways related to mucosal inflammation, antigen processing/presentation, and viral carcinogenesis were downregulated (Figure S3).

### 3.3. Proteomic Findings: Directed Search

**Figure 4:**
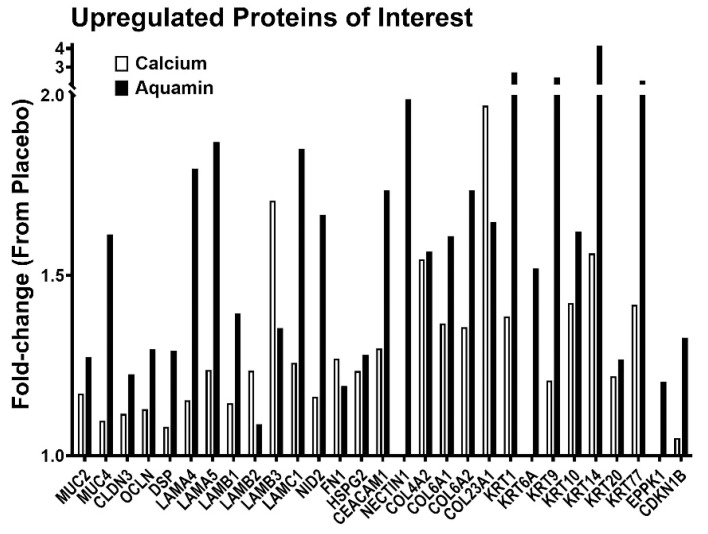
Upregulated proteins of interest. A fold-change value of each protein’s abundance ratio is presented in response to Aquamin^®^ and calcium interventions by comparing these to placebo. This pooled proteomic analysis was based on *n* = 10 subjects in each cohort.

We next searched the database for differentiation-related proteins (keratins) along with proteins involved in cell–cell and cell-matrix adhesion (adherens junction, tight junction, and desmosomal proteins) and other moieties that contribute to barrier structure/function in the colon. These include basement membrane components, carcinoembryonic antigen-related cell adhesion molecules (CEACAMs), mucins, Nectin1, a cell adhesion molecule, and collagen chains. As can be seen from Figure 4, substantial up-regulation (compared to placebo) was observed with many of these proteins in biopsies from subjects ingesting either Aquamin^®^ or calcium alone. For the majority of proteins shown in Figure 4, up-regulation with Aquamin^®^ was greater than with calcium alone.

Most of the identified moieties are epithelial cell products. However, treatment with Aquamin^®^ and/or calcium alone also up-regulated the expression of several collagen chains. Altered expression of collagen chains was not prominent in our earlier colonoid studies [23,24] but seen here; reflecting the fact that the colon biopsies have both epithelial and stromal components. Certain collagen chains (Collagen IV) are integral to basement membrane and others mediate cell attachment to basement membrane [44,45]. The Reactome database was used to identify pathways affected by the proteins up-regulated in response to Aquamin^®^ or calcium alone (Table 1). Not surprisingly, pathways involved in cellular adhesive functions, laminin interactions, and extracellular matrix organization were among the most highly affected.

**Figure 5:**
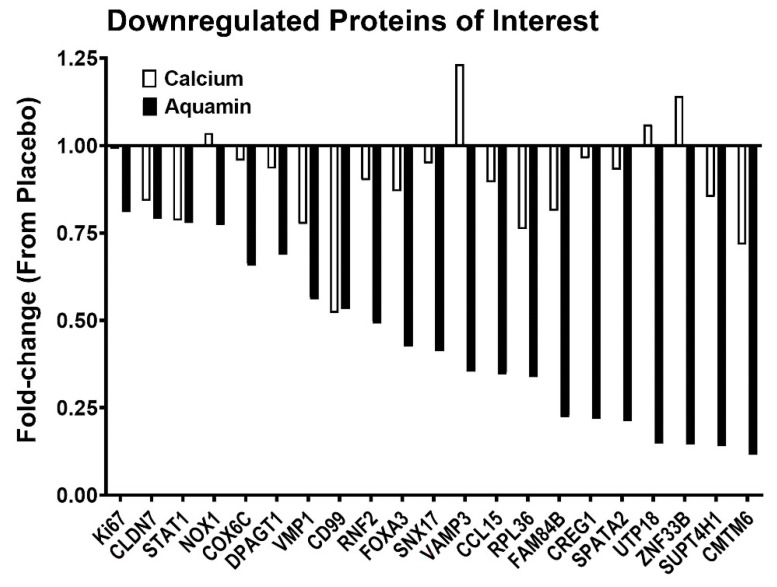
Downregulated proteins of interest. A fold-change value of each protein’s abundance ratio is presented in response to Aquamin^®^ and calcium interventions by comparing these to placebo. This pooled proteomic analysis was based on *n* = 10 subjects in each cohort.

The database was also searched for proteins involved in proliferation, DNA synthesis, tumor metastasis, Wnt/β-catenin signaling, and inflammation. The overexpression of some of these proteins is associated with certain cancers. Several such moieties were identified. Most of these were down-regulated (Figure 5) with Aquamin^®^. Interestingly, many of the growth-related proteins that were decreased in response to Aquamin^®^ were not substantially altered with calcium alone. File S1 provides relevance and further information (including citations) related to the proteins in Figure 5. Finally, we also searched for apoptosis- and calcium metabolism-related moieties. Several of these proteins were upregulated with Aquamin^®^. These are presented in Table S6. These moieties are regulating apoptosis either by inducing or inhibiting apoptosis or programmed cell death. Moieties involved in regulating apoptosis by inhibiting were CARD16, BIRC6, MIEN1, and NOL3. Pro-apoptotic moieties found were IRF3, PPM1F, CYLD, CASP14, BAD, RTN3, PAWR, MAP3K7, MRPL41, and BID. Calcium metabolism-related proteins (CAPN14, PKIG, CRACR2A, PPP3CB, MCUR1, CPPED1, ATP2B4, and CALCOCO2) were involved in calcium binding or transduction of intracellular Ca^2+^-mediated signaling and intracellular trafficking.

## 4. Discussion

We recently completed a 90-day interventional trial in which Aquamin^®^, a calcium-, magnesium-, and trace element-rich multi-mineral natural product derived from red marine algae, was shown to be well-tolerated by healthy adult individuals [35]. The same study demonstrated potentially beneficial effects of Aquamin^®^ based on alterations in gut microbial composition and attendant metabolomic profile. The microbial and metabolomic effects were not observed in a placebo-treated cohort or in subjects treated with calcium alone at an equivalent amount to that provided with Aquamin^®^. Additional findings from the same interventional trial are reported in the current manuscript. Here, we show that while histological features (overall colonic crypt appearance and crypt length) demonstrated no change in histologically-normal colon biopsies from Aquamin^®^-treated subjects relative to those receiving placebo. Meanwhile, a marker of proliferation (Ki67) was reduced with Aquamin^®^ treatment and immunohistochemical markers of cell differentiation (CK20) and (p21) were increased. Consistent with findings of others, calcium alone did not induce a measurable decrease in Ki67 expression [36], but induced increased expression of p21 [36,37].

In parallel with the histological/quantitative immunohistological findings, we utilized a proteomic approach to assess the effects of Aquamin^®^ or calcium alone on protein profile in the colonic mucosa. The data generated in the proteomic screen showed that the 90-day treatment with Aquamin^®^ was sufficient to demonstrate a response, and the number of proteins up- and down-regulated was greater with Aquamin^®^ than the number affected in the calcium group. Among up-regulated proteins, there were several that define the differentiated state and others that contribute to cell–cell and cell–matrix adhesion functions (see Figure 3 and Figure 4). Among down-regulated proteins were Ki67 and several other moieties that contribute to growth-regulation (Figure 5). Additionally, a protein similar to p21 known as CDKN1B or p27 was upregulated with Aquamin^®^. It is a regulator of cell cycle progression [46].

In addition to the upregulated proteins, the proteomic screen uncovered several interesting moieties that were downregulated with Aquamin^®^ (shown in Figure 5 and discussed in File S1). With several of these, overexpression plays a role in proliferation, cell cycle progression, chromatin organization, energy metabolism, and inflammation, as well as tumor invasion and metastasis. For example, the knockdown of a protein known as E3 ubiquitin-protein ligase RING2 (RNF2) inhibits cell proliferation and elevates p21 levels [47]. Similarly, CD99 antigen (CD99) is a cell adhesion molecule and involved in leukocyte transendothelial migration. It is correlated positively with active inflammatory bowel disease (IBD) disease activity [48]. CD99 is also expressed by tumor cells and has a role in tumor progression and cancer cell transendothelial migration [49]. CD99 was also downregulated in our previous study with colonoid culture [24]. Another inflammation-related moiety, C-C motif chemokine 15 (CCL15) is a chemotactic factor that attracts T-cells and monocytes and is involved in inflammation. Loss of SMAD4 promotes CCL15 expression in colon cancer cells and enables primary tumor invasion and liver metastasis of CRC [50]. Interestingly, SMAD4 was upregulated with Aquamin^®^ (1.25-fold) while CCL15 was downregulated (to 0.35-fold).

Additionally, when we compared proteomic profiling of adenomas [22], to the proteomic expression of colon biopsies, there were some moieties (ATP5J2, DPAGT1, PTPLB, HERC1, and COX6C) that were down-regulated in both. The overexpression of ATP5J2 is correlated with enhanced cell migration and decreased 5-FU sensitivity in CRC [51] and the overexpression of the DPAGT1 inhibits E-cadherin’s adhesive function and disrupts tumor cell cohesion [52]. PTPLB is an integral protein of the endoplasmic reticulum membrane [53] and PTPLB turns over rapidly through degradation by the proteasome system and has been associated with metastatic CRC [54]. HERC1, an ubiquitin ligase protein, regulates cell migration via p38 signaling and this regulation is mediated by the mitogen-activated protein kinases (MAPK) kinase MKK3 [55]. COX6C is a component of cytochrome c oxidase, an enzyme in the mitochondrial electron transport chain which drives oxidative phosphorylation and many diseases are linked to the abnormal level of COX6C [56]. Aquamin^®^ also upregulated IRF3 in the colon tissue. It has been documented that the deficiency of IRF3 promotes cellular proliferation in intestinal epithelium and IRF3 protects against colonic tumorigenesis in an AOM/DSS mouse model [57]. Taken together, these findings are potentially significant from a number of standpoints, as discussed below.

The data presented here and in our recent report [35] from the same trial show that a 90-day course of daily treatment with Aquamin^®^ providing 800 mg of calcium per day is sufficient to obtain a measurable effect on markers of differentiation and proliferation in the colonic mucosa as well as to modulate gut microbiome/metabolomic parameters. The effects observed with Aquamin^®^ were not duplicated with calcium alone (though calcium alone did, in fact, alter expression of some of the same markers as Aquamin^®^). Consistent with these findings, our past long-term studies in rodents demonstrated multiple health benefits with Aquamin^®^ (including reduced colon polyp incidence [26,27]) as well as reduced liver injury [58], improved bone mineralization [59,60,61], and reduced inflammatory skin disease [62]) that were only partially mimicked with calcium alone. While our studies with human colon tissue in organoid culture [22,23] also provided evidence for beneficial activity with Aquamin^®^ not seen with calcium alone. Together, these observations support continuation of efforts to demonstrate the utility of a multi-mineral approach for chronic disease prevention and continuation of drug-development efforts with Aquamin^®^ itself.

Regarding relevance to colon cancer, Ki67 is a critical moiety. It is expressed throughout the cell cycle in proliferating cells. A high Ki67 expression is correlated with poor survival, indicating its prognostic value in colorectal cancer patients [63,64]. One of the main findings from this study was a decrease in Ki67-expressing (proliferating) cells as seen by quantitative immunohistology as well as downregulation of Ki67 protein (in proteomic profile). In both platforms, Aquamin^®^’s response in reducing Ki67 was approximately 20%. Ki67 expression was not altered by calcium alone. At the same time, CK20 expression was slightly increased (7%) with Aquamin^®^. Interestingly, there was a striking correlation between findings presented here and results from earlier studies in the colon organoid model [22,23]. In both the normal colon tissue organoid model [23] and the biopsies from the interventional trial (current study), we saw a modest reduction (19% and 20%, respectively) in the proliferation marker (Ki67) without a major change in the differentiation marker (CK20) by immunohistology. In contrast, there was a much larger drop (51%) in the Ki67 expression with Aquamin^®^ with colon adenomas (polyps) in organoid cultures, where the overall positivity was much higher [22]. Similarly, CK20 expression was enhanced in the adenomas with Aquamin^®^. It is clear from these data that adenomas have a higher proliferative index (Ki67 expression) and greater response to Aquamin^®^ than what was seen in normal colon tissue [22,23,65]. In all of these models, Aquamin^®^’s response was much better than calcium’s response, indicating that calcium in conjunction with additional trace minerals has more potential to regulate proliferation and induce differentiation. These findings warrant additional studies to investigate the role of Aquamin^®^ in the prevention of CRC in a long-term polyp prevention trial.

Which trace elements are critical to Aquamin^®^’s chemopreventive activity and the underlying mechanism(s) of action are not fully understood. The importance of calcium cannot be over-estimated, of course, since calcium is the major constituent of Aquamin^®^, and epidemiological studies have clearly demonstrated a strong relationship between calcium intake and reduced colon tumor incidence [6,7,8,9,10,11]. Animal studies have directly demonstrated inhibition of colon tumor formation with calcium [26,27,28], and in vitro studies have provided mechanistic insight into how calcium modulates epithelial growth [29]. Past studies have shown that activation of the extracellular calcium-sensing receptor (CaSR) on the epithelial cell surface is critical to growth control [21,66,67,68]. This is of interest because several of the trace elements present in Aquamin^®^, including members of the lanthanoid family, have a higher affinity for CaSR than calcium itself [69]. The combination of calcium and lanthanoid can increase calcium-dependent biological responses over that seen with calcium alone [70,71]. Thus, one of the mechanisms through which Aquamin^®^ might produce better growth control than calcium alone is through enhanced activation of CaSR. Other mechanisms are also possible. It has been shown, for example, that the ratio of magnesium to calcium is as important as the level of calcium for growth control. A molar ratio close to that found in Aquamin^®^ is beneficial [19]. Finally, other minerals present in Aquamin^®^ have been shown to independently affect tumor formation in experimental models [72,73]. Perhaps it is the presence of multiple anti-tumor mechanisms that underlie the anti-tumor efficacy of the natural product.

Although the major focus of this effort was colon polyp prevention, our findings allow us to suggest that benefits of multi-mineral intervention may extend beyond this indication. Specifically, the up-regulation of multiple adhesion molecules, both in the interventional model described here and in organoid culture [22,23,24,25], may translate into improved colon mucosal barrier. In the organoid culture model, it should be noted, exposure to Aquamin^®^ resulted in increased transepithelial electrical resistance and increased tissue cohesion in parallel with adhesion molecule and barrier protein up-regulation [25]. Defects in barrier function–allowing gut bacteria, food allergens, and potentially toxic substances access to the interstitial space–are likely to contribute to inflammatory bowel diseases (IBD) including Crohn’s disease and ulcerative colitis [74,75]. A loss of permeability control has also been noted in relation to irritable bowel syndrome [76]. More recently, it has been suggested that chronic systemic inflammation associated with obesity occurs, in part at least, as a consequence of high fat diet-induced defects in gastrointestinal permeability control [75]. While separating cause and effect in these various conditions is not straightforward, an improvement in barrier function would seem to be worthwhile in any case. Similarly, optimal gut barrier improvement may protect against CRC as well as inflammatory diseases [77]. Improved barrier function with a low-cost, low (to no)- toxicity agent could provide a cost-effective adjuvant therapy for a large group of individuals who suffer from chronic (and painful) inflammatory bowel conditions associated with barrier defects. Whether this will work, ultimately, and to what extent needs to be established experimentally in a carefully controlled clinical trial. To this end, we have recently initiated a 180-day, phase I trial (Clinicaltrials.gov; NCT03869905), evaluating the efficacy of Aquamin^®^ as adjuvant therapy for individuals with ulcerative colitis in remission. Results from the recently-finished trial along with our organoid culture data and the results presented here should together provide an indication as to whether a multi-mineral approach to barrier improvement in the colon is helpful.

The findings presented here are promising, but must be taken as preliminary since the number of subjects (10 per group) was small and the treatment period (90 days) was short. For example, we observed a decrease (20%) in Ki67 expression and increase (99%) in p21 expression with Aquamin^®^ in post-intervention biopsies from the baseline expression, however, this change was not significant when analyzed by subject number in each group (*n* = 10). When we evaluated these data based on the number of crypts analyzed per group, both trends became statistically significant (*p* and *q* values < 0.05). Another limitation: we simply do not know if the biomarker changes reported here with Aquamin^®^ will, ultimately, be reflective of a reduction in colon polyp incidence or with any other therapeutic benefit. One more limitation is that we used Aquamin^®^, a product that contains calcium and magnesium along with multiple trace minerals in the current studies. The findings presented here and those described earlier suggest the importance of multiple minerals as a combination [12,13,14,20,21,22,23,24,25,26,27,35,58,59,60,61,62,71]. However, additional studies are needed to identify the subset of minerals that are critical. The findings are sufficiently encouraging, we believe, to support further effort. As noted above, a therapeutic trial for 180 days (with 20 subjects in each arm) is currently underway. Equally important, while the findings here are based on results from a small number of subjects, our findings may be of value in so far as they could help other investigators by providing an indication of how large a change might be expected in several colonic biomarkers.

## 5. Conclusions

In summary, while it is well-known that having an adequate daily calcium intake is important for preventing various chronic, long-latency diseases [1,2,3,4,5] including colorectal cancer [6,7,8,9,10,11], recent epidemiological studies have begun to appreciate the role of trace elements other than calcium in the mitigation of chronic diseases [12,13,14,15]. While our past preclinical studies [20,21,22,23,24,25,26,27] have provided compelling evidence that a multi-mineral approach with a natural product such as Aquamin^®^ can provide benefit not seen with calcium alone, results presented in our recent report [35] and those described here suggest that responses to multi-mineral intervention (as a potential chemopreventive agent) are robust enough to be seen in a small and comparatively simple clinical interventional trial setting.

## Figures and Tables

**Table 1:** Significantly altered pathways (by upregulated proteins with directed search).

Pathway Name	Entities *p* Value	Entities FDR	Mapped Entities
Laminin interactions	1.1 × 10^−16^	1.9 × 10^−14^	COL4A2; HSPG2; LAMA4; LAMA5; LAMB1; LAMB2; LAMB3; LAMC1; NID2
ECM proteoglycans	3.0 × 10^−15^	2.3 × 10^−13^	COL4A2; COL6A1; COL6A2; FN1; HSPG2; LAMA4; LAMA5; LAMB1; LAMB2; LAMC1
Extracellular matrix organization	4.1 × 10^−15^	2.3 × 10^−13^	CEACAM1; COL4A2; COL6A1; COL6A2; COL23A1; FN1; HSPG2; LAMA4; LAMA5; LAMB1; LAMB2; LAMB3; LAMC1; NID2
Non-integrin membrane-ECM interactions	2.4 × 10^−14^	9.9 × 10^−13^	COL4A2; FN1; HSPG2; LAMA4; LAMA5; LAMB1; LAMB2; LAMB3; LAMC1
Degradation of the extracellular matrix	1.2 × 10^−12^	3.6 × 10^−11^	COL4A2; COL6A1; COL6A2; COL23A1; FN1; HSPG2; LAMA5; LAMB1; LAMB3; LAMC1
MET activates PTK2 signaling	1.3 × 10^−12^	3.6 × 10^−11^	FN1; LAMA4; LAMA5; LAMB1; LAMB2; LAMB3; LAMC1
MET promotes cell motility	1.1 × 10^−11^	2.7 × 10^−10^	FN1; LAMA4; LAMA5; LAMB1; LAMB2; LAMB3; LAMC1
Formation of the cornified envelope	9.1 × 10^−10^	1.9 × 10^−8^	DSP; KRT1; KRT10; KRT14; KRT20; KRT6A; KRT9; KRT77
Signaling by MET	1.1 × 10^−9^	2.0 × 10^−8^	FN1; LAMA4; LAMA5; LAMB1; LAMB2; LAMB3; LAMC1
Keratinization	5.0 × 10^−8^	8.1 × 10^−7^	DSP; KRT1; KRT6A; KRT9; KRT10; KRT14; KRT20; KRT77
Integrin cell surface interactions	6.9 × 10^−8^	1.0 × 10^−6^	COL4A2; COL6A1; COL6A2; COL23A1; FN1; HSPG2
Signaling by Receptor Tyrosine Kinases	3.6 × 10^−7^	5.0 × 10^−6^	COL4A2; COL6A1; COL6A2; FN1; LAMA4; LAMA5; LAMB1; LAMB2; LAMB3; LAMC1
Developmental Biology	1.0 × 10^−6^	1.2 × 10^−5^	COL4A2; COL6A1; COL6A2; DSP;KRT1; KRT10; KRT14; KRT20; KRT6A; KRT9; KRT77; LAMB1; LAMC1
Collagen formation	3.1 × 10^−6^	3.7 × 10^−5^	COL23A1; COL4A2; COL6A1; COL6A2; LAMB3
Collagen chain trimerization	4.8 × 10^−6^	5.3 × 10^−4^	COL23A1; COL4A2; COL6A1; COL6A2
Assembly of collagen fibrils and other multimeric structures	1.7 × 10^−5^	1.7 × 10^−4^	COL4A2; COL6A1; COL6A2; LAMB3
Collagen degradation	2.1 × 10^−5^	1.9 × 10^−4^	COL23A1; COL4A2; COL6A1; COL6A2
Collagen biosynthesis and modifying enzymes	2.5 × 10^−5^	2.2 × 10^−4^	COL23A1; COL4A2; COL6A1; COL6A2
Cell junction organization	8.5 × 10^−5^	0.001	CLDN3; KRT14; LAMB3; NECTIN1
Fibronectin matrix formation	1.1 × 10^−4^	0.001	CEACAM1; FN1
Post-translational protein phosphorylation	1.5 × 10^−4^	0.001	FN1; LAMB1; LAMB2; LAMC1
NCAM1 interactions	1.7 × 10^−4^	0.001	COL4A2; COL6A1; COL6A2
Regulation of IGF Factor transport and uptake by IGFBPs	2.6 × 10^−4^	0.002	FN1; LAMB1; LAMB2; LAMC1
Cell-Cell communication	3.2 × 10^−4^	0.002	CLDN3; KRT14; LAMB3; NECTIN1
Type I hemidesmosome assembly	3.7 × 10^−4^	0.002	KRT14; LAMB3
Apoptotic cleavage of cell adhesion proteins	3.7 × 10^−4^	0.002	DSP; OCLN
Signaling by PDGF	4.8 × 10^−4^	0.003	COL4A2; COL6A1; COL6A2
NCAM signaling for neurite out-growth	5.7 × 10^−4^	0.003	COL4A2; COL6A1; COL6A2
Anchoring fibril formation	6.8 × 10^−4^	0.003	COL4A2; LAMB3
Defective GALNT12 causes colorectal cancer 1	0.001	0.005	MUC2; MUC4
Termination of O-glycan biosynthesis	0.002	0.01	MUC2; MUC4
Apoptotic cleavage of cellular proteins	0.004	0.02	DSP; OCLN
Apoptotic execution phase	0.01	0.03	DSP; OCLN
Dectin-2 family	0.01	0.04	MUC2; MUC4
O-linked glycosylation of mucins	0.01	0.04	MUC2; MUC4
Cell-cell junction organization	0.01	0.04	CLDN3; NECTIN1
RUNX1 regulates expression of components of tight junctions	0.01	0.04	OCLN
PTK6 Regulates Cell Cycle	0.02	0.05	CDKN1B
Nectin/Necl trans heterodimerization	0.02	0.05	NECTIN1

These altered pathways are involved with the set of up-regulated proteins presented in Figure 4. Reactome (v74) was used to generate the pathway analysis report for species “Homo sapiens”. These significant data (with *p*-value/FDR < 0.05) are based on the overrepresentation analysis (hypergeometric distribution). Insulin-like growth factor: IGF; insulin-like growth factor binding protein: IGFBP; neural cell adhesion molecule: NCAM.

## Data Availability

The raw mass spectrometry proteomics data are available on ProteomeXchange Consortium (PRIDE partner repository)–identifier: PXD024445.

## Supplementary Materials

The following are available online at https://www.mdpi.com/2072-6643/13/3/939/s1, Figure S1: Change in histological and immunohistological features, Figure S2: Unbiased proteomic screen: Protein interactions, Figure S3: Unbiased proteomic screen: Altered pathways, Table S1: Amount of Minerals and Trace Elements in a daily dose of Aquamin^®^ TG, Table S2: List of antibodies used for quantitative immunohistochemistry (qIHC), Table S3: Unbiased approach: Upregulated moieties, Table S4: Unbiased approach: Downregulated moieties, Table S5: Significantly enriched GO Biological Processes; Significantly enriched GO Molecular Functions; Significantly enriched GO Cellular Components, Table S6: Additional proteins of interest, Supplemental File S1. Downregulated Proteins of Interest (Proteins presented in Figure 5).
