# Supplementary material for: A Multi-Mineral Intervention to Modulate Colonic Mucosal Protein Profile: Results from a 90-Day Trial in Human Subjects
Source: Nutrients. 2021 Mar 14;13(3):939. doi: 10.3390/nu13030939 (PMC8002192; doi:10.3390/nu13030939)
Supplement: Supplementary file 1 [file nutrients-13-00939-s001.zip › Figure S1_Nutrients-1121446.pdf]

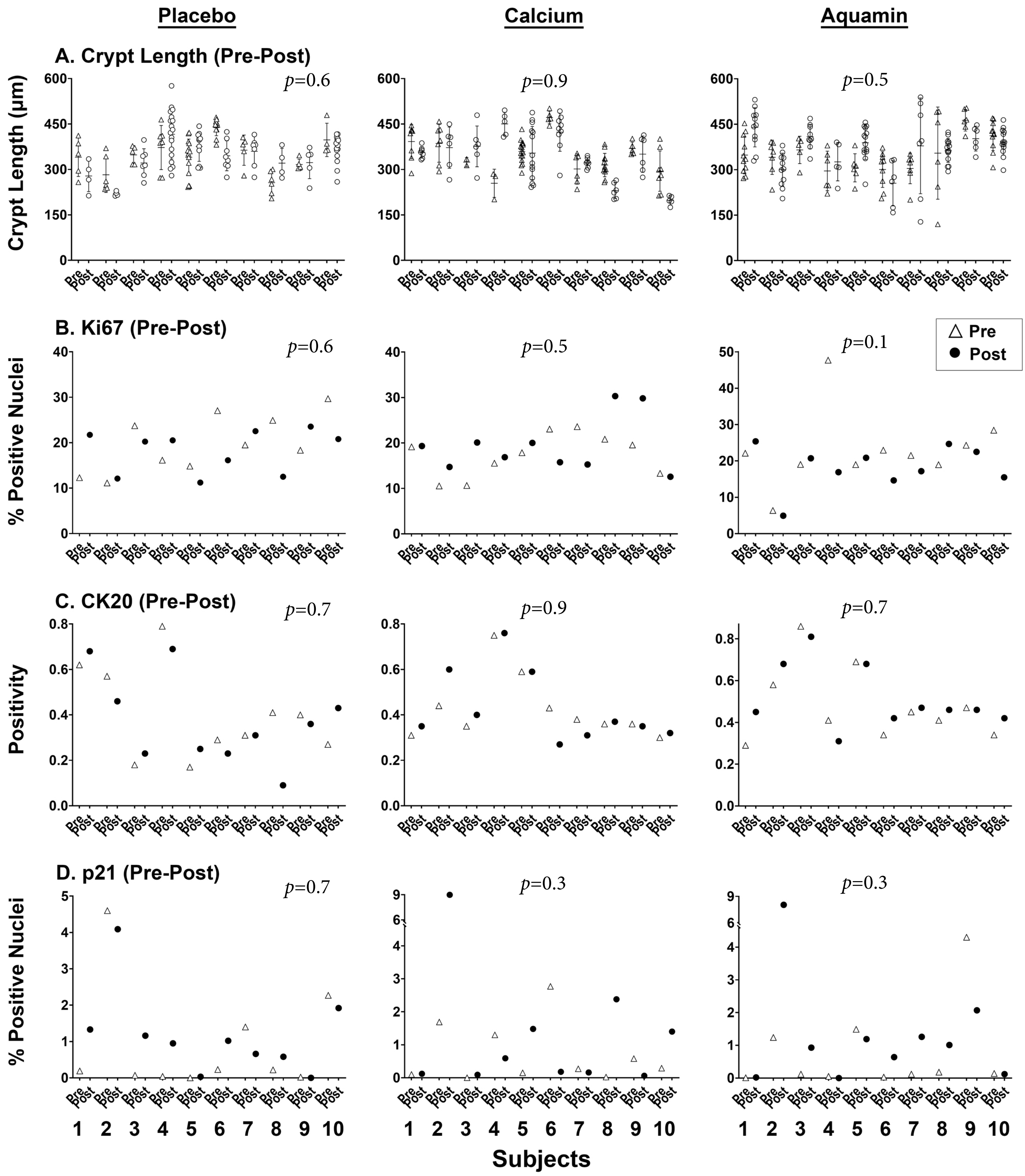

Placebo
Calcium
Aquamin
A. Crypt Length (Pre-Post)
Crypt Length (µm)
p=0.6
p=0.9
p=0.5
B. Ki67 (Pre-Post)
% Positive Nuclei
p=0.6
p=0.5
p=0.1
Pre
Post
C. CK20 (Pre-Post)
Positivity
p=0.7
p=0.9
p=0.7
D. p21 (Pre-Post)
% Positive Nuclei
p=0.7
p=0.3
p=0.3
Subjects
