# Supplementary material for: A Multi-Mineral Intervention to Modulate Colonic Mucosal Protein Profile: Results from a 90-Day Trial in Human Subjects
Source: Nutrients. 2021 Mar 14;13(3):939. doi: 10.3390/nu13030939 (PMC8002192; doi:10.3390/nu13030939)

Protein-Protein Interactions: Unbiased Approach (1.5-fold & FDR ≤1%).

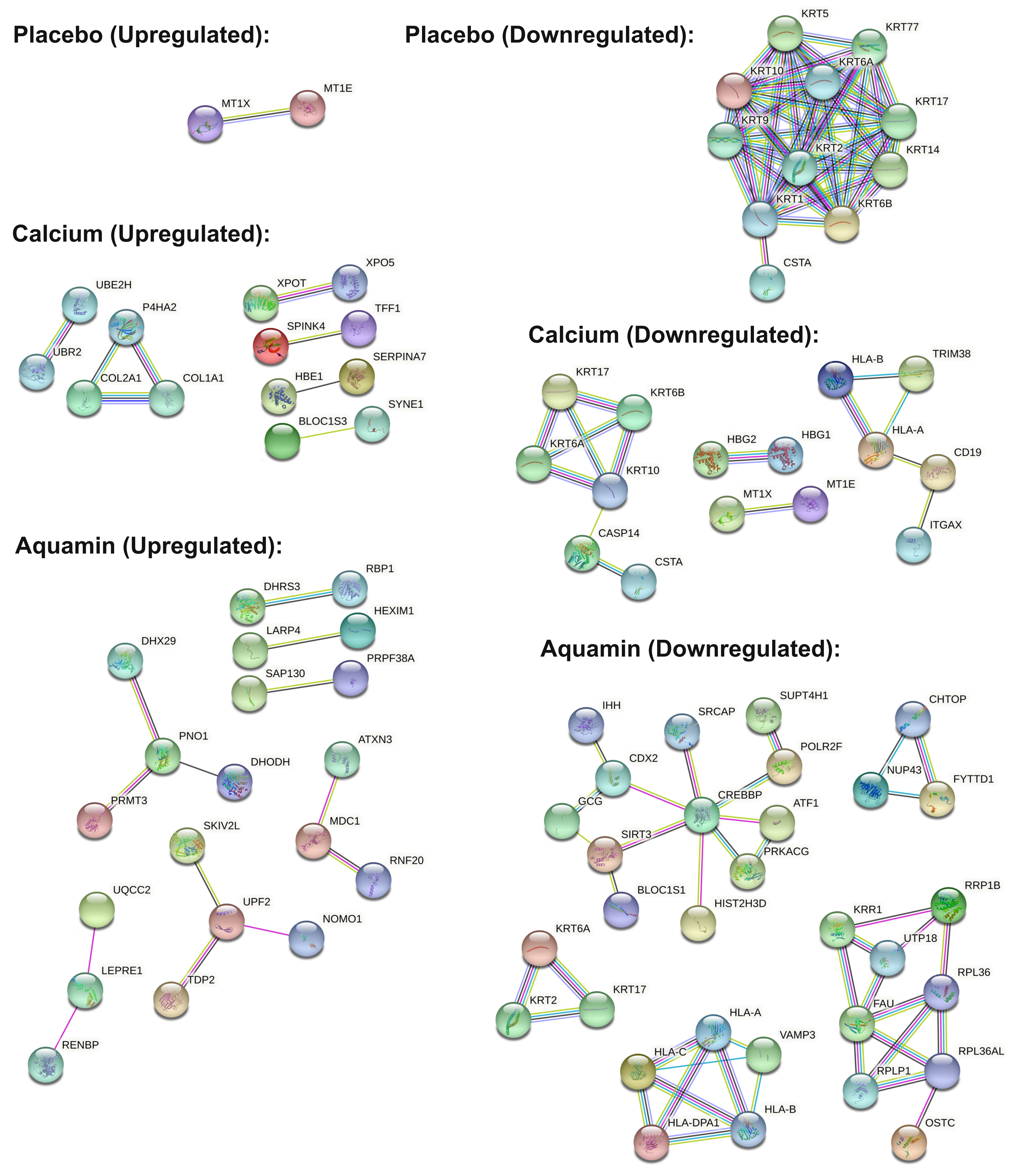

Supplement: Supplementary file 1 [file nutrients-13-00939-s001.zip › Figure S2_Nutrients-1121446.pdf]
