# Supplementary material for: A Multi-Mineral Intervention to Modulate Colonic Mucosal Protein Profile: Results from a 90-Day Trial in Human Subjects
Source: Nutrients. 2021 Mar 14;13(3):939. doi: 10.3390/nu13030939 (PMC8002192; doi:10.3390/nu13030939)
Supplement: Supplementary file 1 [file nutrients-13-00939-s001.zip › Figure S3_Nutrients-1121446.pdf]

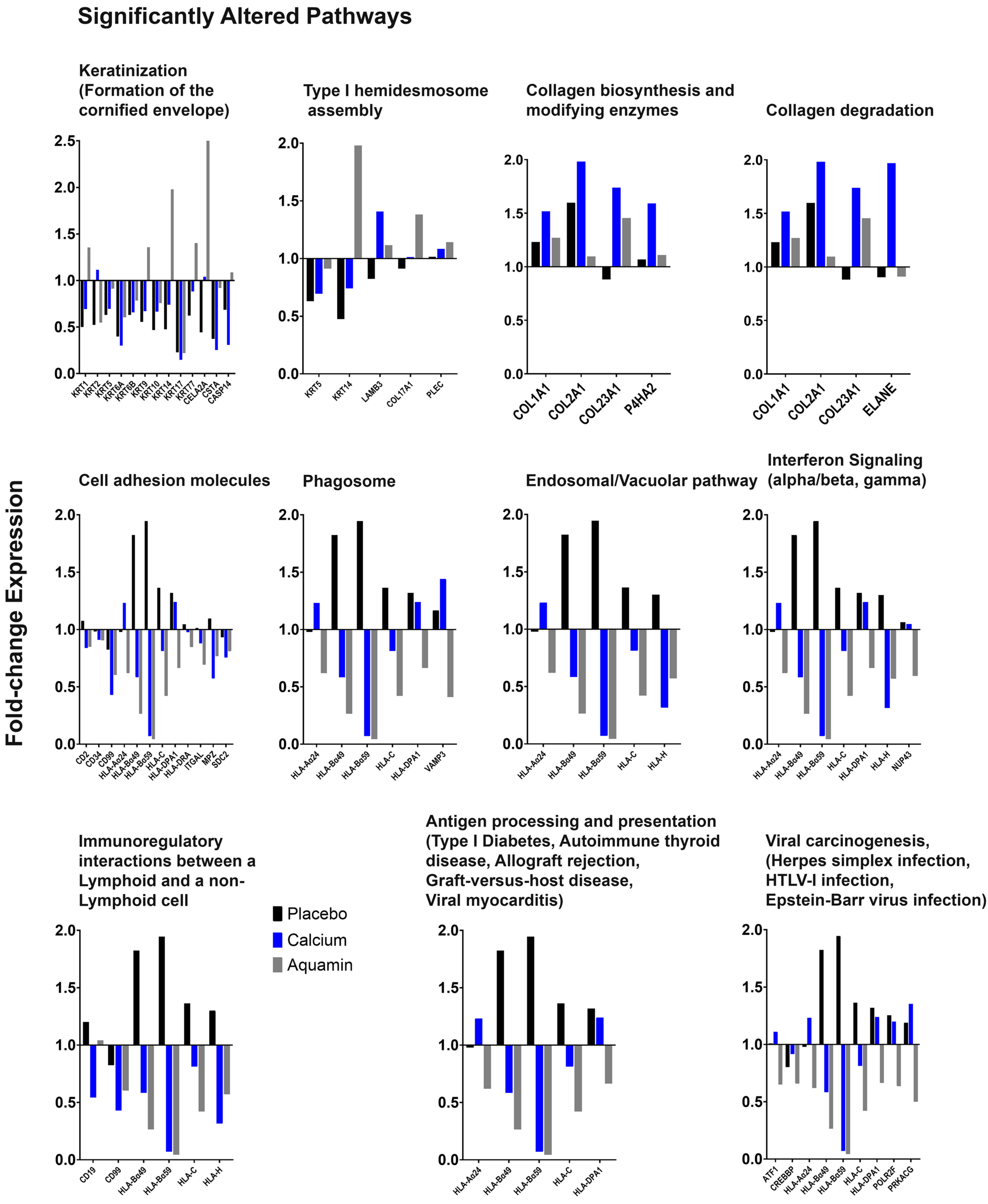

Significantly Altered Pathways
Fold-change Expression
Keratinization (Formation of the cornified envelope)
KRT1 KRT2 KRT5 KRT6A KRT6B KRT9 KRT10 KRT14 KRT17 KRT77 CELA2A CSTA CASP14
Type I hemidesmosome assembly
KRT5 KRT14 LAMB3 COL17A1 PLEC
Collagen biosynthesis and modifying enzymes
COL1A1 COL2A1 COL23A1 P4HA2
Collagen degradation
COL1A1 COL2A1 COL23A1 ELANE
Cell adhesion molecules
CD2 CD34 CD99 HLA-Aα24 HLA-Bα49 HLA-Bα59 HLA-C HLA-DPA1 HLA-DRA ITGAL MPZ SDC2
Phagosome
HLA-Aα24 HLA-Bα49 HLA-Bα59 HLA-C HLA-DPA1 VAMP3
Endosomal/Vacuolar pathway
HLA-Aα24 HLA-Bα49 HLA-Bα59 HLA-C HLA-H
Interferon Signaling (alpha/beta, gamma)
HLA-Aα24 HLA-Bα49 HLA-Bα59 HLA-C HLA-DPA1 HLA-H NUP43
Immunoregulatory interactions between a Lymphoid and a non-Lymphoid cell
CD19 CD99 HLA-Bα49 HLA-Bα59 HLA-C HLA-H
Placebo
Calcium
Aquamin
Antigen processing and presentation (Type I Diabetes, Autoimmune thyroid disease, Allograft rejection, Graft-versus-host disease, Viral myocarditis)
HLA-Aα24 HLA-Bα49 HLA-Bα59 HLA-C HLA-DPA1
Viral carcinogenesis, (Herpes simplex infection, HTLV-I infection, Epstein-Barr virus infection)
ATF1 CREBBP HLA-Aα24 HLA-Bα49 HLA-Bα59 HLA-C HLA-DPA1 POLR2F PRKACG
