# Supplementary material for: A Multi-Mineral Intervention to Modulate Colonic Mucosal Protein Profile: Results from a 90-Day Trial in Human Subjects
Source: Nutrients. 2021 Mar 14;13(3):939. doi: 10.3390/nu13030939 (PMC8002192; doi:10.3390/nu13030939)
Supplement: Supplementary file 1 [file nutrients-13-00939-s001.zip › Supplementary Figure Legends.pdf]

Supplementary Figure Legends.

Figure S1. Change in histological and immunohistological features. The pre-post difference in the colonic mucosa of each subject after 90 days of intervention. Pre- post-intervention group averages and standard deviations were used to calculate *p* values (by two-stage linear step-up procedure of Benjamini, Krieger and Yekutieli) for each feature. (n=10 subjects per group).

A: Crypt length, **B**: Ki67, **C**: CK20, **D**: p21.

Figure S2. Unbiased proteomic screen: Protein interactions. A network view of protein-protein interactions and predicted associations for the group of proteins at 1.5-fold change; ≤1% FDR (presented in Figure 3 and listed in Table S3 and Table S4) by employing STRING-database (v11). The strong interactions or associations can be seen with thick connecting lines. These lines are color-coded. Red line—indicates the presence of fusion evidence; Green line—neighborhood evidence; Blue line—co-occurrence evidence; Purple line—experimental evidence; Yellow line—text mining evidence; Light blue line—database evidence; Black line—co-expression evidence. There are multiple distinct clusters of proteins that are evident. The most prominent is in the placebo group with a set of downregulated proteins (Keratins & Cystatin-A [CSTA]).

Figure S3. Unbiased proteomic screen: Altered pathways. Most significant pathways with the altered proteins at 1.5-fold change (presented in Figure 3 and listed in Table S3 and Table S4). String database (v11) was used for the enrichment analysis to generate altered pathways (KEGG and Reactome (v74) were the source databases for the pathways).
