# Supplementary material for: A Multi-Mineral Intervention to Modulate Colonic Mucosal Protein Profile: Results from a 90-Day Trial in Human Subjects
Source: Nutrients. 2021 Mar 14;13(3):939. doi: 10.3390/nu13030939 (PMC8002192; doi:10.3390/nu13030939)
Supplement: Supplementary file 1 [file nutrients-13-00939-s001.zip › Table S1_Nutrients-1121446.pdf]

Table S1. Amount of Minerals and Trace Elements present in a daily dose of Aquamin® TG

Element	μg/day	Element	μg/day	Element	μg/day
Aluminum	488	Hafnium	0.1	Rubidium	0.04
Antimony	<1.0	Holmium	0.03	Ruthenium	5.3
Arsenic	2.0	Indium	<0.002	Samarium	0.1
Barium	11	Iodine	45	Scandium	2.4
Beryllium	<1.0	Iridium	<0.002	Selenium	<1.0
Bismuth	<1.0	Iron	1617	Silicon	256
Boron	81	Lanthanum	<1.0	Silver	7.2
Cadmium	1.2	Lead	0.2	Sodium	11666
Calcium	800000	Lithium	<1.0	Strontium	5315
Carbon	300381	Lutetium	0.04	Sulfur	8340
Cerium	0.7	Magnesium	33300	Tantalum	0.04
Cesium	0.002	Manganese	87	Tellurium	<1.0
Chloride	2039	Mercury	<0.002	Terbium	0.02
Chromium	<1.0	Molybdenum	<1.0	Thallium	<1.0
Cobalt	1.4	Neodymium	0.4	Thorium	16
Copper	4.7	Nickel	<1.0	Thulium	0.01
Dysprosium	0.1	Niobium	<1.0	Tin	0.2
Erbium	0.1	Osmium	<0.002	Titanium	74
Europium	0.03	Palladium	0.6	Tungsten	<1.0
Fluoride	2.4	Phosphorous	1312	Vanadium	6.4
Gadolinium	0.1	Platinum	0.01	Ytterbium	0.1
Gallium	0.3	Potassium	793	Yttrium	<1.0
Germanium	<0.002	Praseodymium	0.1	Zinc	27
Gold	10	Rhenium	0.005	Zirconium	12
		Rhodium	0.4		

These amounts were calculated from the mineral composition of Aquamin® TG used in this phase I trial. The mineral composition was measured by an independent laboratory (Advanced Laboratories, Inc. Salt Lake City) for client Marigot Limited (Ireland) in 2015.
Most trace elements were determined by Inductively Coupled Plasma Optical Emission Spectrometry (ICP-OES) except for Carbon (determined by LECO), Chloride, Iodine (determined by Titration), and Fluoride (determined by AOAC 939.11).
