# Supplementary material for: A Multi-Mineral Intervention to Modulate Colonic Mucosal Protein Profile: Results from a 90-Day Trial in Human Subjects
Source: Nutrients. 2021 Mar 14;13(3):939. doi: 10.3390/nu13030939 (PMC8002192; doi:10.3390/nu13030939)
Supplement: Supplementary file 1 [file nutrients-13-00939-s001.zip › Table S2_Nutrients-1121446.pdf]

Table S2. List of antibodies used for quantitative immunohistochemistry (qIHC)

Antibody	Vendor	Catalog #	Dilution	Incubation Time	Retrieval Method
Rb Ki-67 mAb clone SP6	Cell Marque	275R-16	1:250	30 min	[a]HIER pH 9.0, 20 min
Ms CK20 mAb clone K_s20.8	Dako	M7019	1:100	60 min	FLEX TRS High pH 9.0, 20 min
Ms p21 (WAF1) mAb clone 4D10	Novocastra (Leica)	NCL-L-WAF-1	1:40	120 min	[a]HIER pH 6.0, 40 min

[a]HIER: Heat induced epitope retrieval 10 mM Tris HCl/1 mM EDTA buffer

These antibodies are used for immunohistochemical (IHC) staining of formalin-fixed, paraffin-embedded (FFPE) colon tissue sections. Positive and negative control tissues (or negative control reagent) ran simultaneously using the same protocol as the actual colon specimens.
