# Supplementary material for: A Multi-Mineral Intervention to Modulate Colonic Mucosal Protein Profile: Results from a 90-Day Trial in Human Subjects
Source: Nutrients. 2021 Mar 14;13(3):939. doi: 10.3390/nu13030939 (PMC8002192; doi:10.3390/nu13030939)
Supplement: Supplementary file 1 [file nutrients-13-00939-s001.zip › Table S3_Nutrients-1121446.pdf]

Table S3. Unbiased approach: Upregulated moieties.
(at 1.5-fold and ≤1%FDR)

Protein (Gene)	Placebo	Calcium	Aquamin	Calcium (Compared to Placebo)	Aquamin (Compared to Placebo)
Common upregulated proteins in all 3 groups (0)					
Common upregulated proteins between Placebo and Calcium groups (1)					
COL2A1	**1.597**	**1.981**	1.096	1.240	0.686
Common upregulated proteins between Calcium and Aquamin groups (2)					
ZBED1	0.991	**1.824**	**1.717**	1.840	1.732
TIMM10B	1.280	**1.703**	**1.518**	1.331	1.186
Common upregulated proteins between Placebo and Aquamin groups (0)					
Upregulated proteins - Unique to Aquamin (68)					
ESRRA	0.366	0.462	**2.632**	1.262	7.195
CELA2A	0.442	1.039	**2.565**	2.354	5.809
HLA-B α37	0.202	1.323	**2.364**	6.560	11.721
SERF2	0.524	0.626	**2.340**	1.194	4.463
PRPF38A	0.719	0.788	**2.188**	1.096	3.043
NDUFAF3	0.952	1.212	**2.182**	1.273	2.293
PMVK	0.896	0.896	**2.146**	1.000	2.395
ATXN3	0.491	0.465	**2.104**	0.949	4.288
NOMO1	0.873	1.366	**2.019**	1.565	2.313
RNF20	0.732	0.790	**2.009**	1.079	2.744
KRT14	0.475	0.741	**1.980**	1.561	4.170
FAAH	0.789	0.742	**1.977**	0.940	2.505
PITPNM1	0.964	0.854	**1.906**	0.886	1.977
DAAM2	0.718	0.906	**1.897**	1.262	2.644
FAM105B	0.752	0.769	**1.835**	1.023	2.439
BAIAP2	0.527	0.840	**1.810**	1.594	3.435
SRGAP2C	0.785	0.767	**1.779**	0.976	2.265
PNO1	0.731	0.870	**1.774**	1.190	2.427
ZNF703	0.746	1.068	**1.774**	1.431	2.377
TMBIM1	0.781	0.825	**1.765**	1.057	2.259
LARP4	1.039	0.918	**1.756**	0.884	1.690
SLC12A7	0.644	0.781	**1.749**	1.212	2.713
SKIV2L	0.833	0.711	**1.747**	0.855	2.098
QTRT2	0.836	0.973	**1.735**	1.164	2.076
BCHE	0.852	0.805	**1.731**	0.946	2.032
FAM109B	0.497	1.112	**1.719**	2.238	3.459
TECPR1	0.908	1.008	**1.710**	1.110	1.883
PACSIN3	0.662	0.799	**1.710**	1.207	2.583
MDC1	0.875	1.210	**1.692**	1.383	1.934
UQCC2	0.797	0.839	**1.684**	1.053	2.113
SLC41A3	1.022	0.849	**1.683**	0.831	1.648
TDP2	0.793	0.867	**1.678**	1.094	2.116
HEXIM1	0.792	0.979	**1.676**	1.236	2.116
SLC39A11	0.536	1.007	**1.675**	1.878	3.122
ATL2	0.707	0.780	**1.674**	1.103	2.369
SKAP2	0.882	1.007	**1.660**	1.142	1.882
FRG1	0.592	0.733	**1.658**	1.238	2.801

DHODH	0.872	0.854	**1.654**	0.980	1.898
UPF2	0.816	0.930	**1.649**	1.139	2.020
BIRC6	0.899	0.958	**1.647**	1.066	1.831
EIF2B2	0.994	0.692	**1.629**	0.696	1.639
DHX29	0.877	0.981	**1.628**	1.119	1.857
FER1L6	0.926	1.023	**1.610**	1.104	1.738
ALDH1L1	0.801	0.813	**1.609**	1.015	2.008
TMEM106B	0.891	1.064	**1.609**	1.194	1.805
DHRS3	0.845	0.785	**1.601**	0.928	1.894
RAB3B	0.857	0.857	**1.590**	1.000	1.856
P3H1	0.730	0.829	**1.588**	1.136	2.175
COX16	0.809	0.885	**1.583**	1.093	1.955
IGKV1-12	0.984	0.850	**1.581**	0.864	1.607
WASH6P	0.728	1.069	**1.577**	1.469	2.166
CHCHD6	0.830	1.061	**1.575**	1.278	1.898
TMSB15B	0.854	0.804	**1.570**	0.941	1.838
PRMT3	1.337	1.134	**1.564**	0.848	1.170
SULT1C2	0.906	0.961	**1.562**	1.060	1.724
HEATR3	0.860	0.921	**1.551**	1.071	1.804
RENBP	1.034	1.000	**1.544**	0.966	1.492
FITM2	0.766	0.851	**1.531**	1.111	1.998
MED20	0.958	0.801	**1.530**	0.835	1.596
CARD16	0.741	0.797	**1.529**	1.074	2.062
SLC35A2	0.541	0.697	**1.527**	1.288	2.822
NCOA6	0.673	0.895	**1.527**	1.329	2.267
ARID2	0.784	0.784	**1.521**	1.000	1.940
NUDT3	0.813	0.765	**1.514**	0.942	1.863
UBAC1	0.829	0.922	**1.505**	1.111	1.815
RBP1	0.931	0.848	**1.503**	0.910	1.614
TMEM222	0.925	0.803	**1.500**	0.868	1.620
SAP130	0.828	0.816	**1.500**	0.985	1.811
	Upregulated proteins - Unique to Calcium (44)				
BLOC1S3	1.174	**2.595**	1.247	2.211	1.062
TXNDC15	1.035	**2.365**	1.004	2.284	0.970
HBE1	0.791	**2.302**	0.954	2.910	1.206
BCR	0.892	**2.185**	1.195	2.451	1.340
CCL21	0.940	**2.163**	1.125	2.300	1.197
RARRES1	0.903	**2.108**	1.341	2.335	1.485
ATRN	0.944	**1.971**	1.248	2.087	1.321
ELANE	0.903	**1.968**	0.909	2.179	1.007
DHX30	0.755	**1.901**	1.125	2.520	1.490
AMY2A	1.172	**1.872**	0.517	1.598	0.441
CROT	1.004	**1.868**	0.831	1.861	0.827
PTBP3	0.936	**1.846**	0.790	1.972	0.844
CAPS	0.919	**1.796**	1.065	1.955	1.159
DNAJC1	0.846	**1.784**	0.802	2.109	0.948
RNMT	0.996	**1.778**	1.127	1.785	1.131
ECHDC3	0.873	**1.777**	1.046	2.035	1.198
HLA-A α31	0.797	**1.773**	0.856	2.224	1.074
SMG5	0.919	**1.732**	1.024	1.885	1.115
CPSF2	1.032	**1.724**	1.071	1.671	1.038

SPINK4	0.997	**1.722**	0.901	1.727	0.904
FAT1	0.937	**1.717**	1.454	1.833	1.552
SYNE1	0.996	**1.686**	1.363	1.694	1.369
DUSP16	0.862	**1.683**	1.039	1.953	1.205
HSP90AA4P	0.969	**1.679**	1.093	1.733	1.128
AKAP5	1.021	**1.661**	1.083	1.627	1.061
PKLR	0.769	**1.654**	1.267	2.151	1.648
XPO5	1.033	**1.634**	1.027	1.582	0.995
METTL13	1.191	**1.605**	1.179	1.347	0.990
WASHC3	1.171	**1.604**	0.885	1.370	0.755
PCIF1	0.892	**1.604**	1.039	1.798	1.165
UBR2	1.011	**1.602**	1.062	1.585	1.050
P4HA2	1.068	**1.590**	1.109	1.488	1.038
ATP8A1	1.026	**1.579**	0.964	1.539	0.940
TSPAN1	1.047	**1.565**	1.033	1.494	0.987
WDR98	1.235	**1.556**	1.166	1.260	0.944
GGT2	1.018	**1.554**	1.313	1.527	1.290
UBE2H	1.228	**1.545**	0.835	1.258	0.680
XPOT	0.977	**1.543**	1.380	1.579	1.413
SERPINA7	1.063	**1.542**	0.750	1.450	0.705
TFF1	1.215	**1.536**	1.062	1.265	0.874
COL1A1	1.230	**1.516**	1.271	1.232	1.033
STAT4	0.970	**1.512**	1.233	1.559	1.272
STAB1	1.003	**1.504**	1.107	1.500	1.104
RFC3	0.847	**1.503**	1.136	1.775	1.341
Upregulated proteins - Unique to Placebo (6)					
HLA-B α59	**1.946**	0.070	0.044	0.036	0.023
HLA-B α49	**1.823**	0.584	0.265	0.320	0.145
MT1E	**1.747**	0.540	1.492	0.309	0.854
SUPT4H1	**1.665**	1.421	0.233	0.853	0.140
MT1X	**1.578**	0.580	0.675	0.368	0.427
CTNNBIP1	**1.521**	1.058	0.748	0.696	0.492

The values represent fold-change of abundance ratio as compared to the control (all baseline samples and post-intervention placebo samples). For each upregulated protein, corresponding values from the other groups are shown for comparison. These samples were assessed by TMT based differential proteomic expression by pooling the samples for each group. Protein FDR Confidence for all the proteins was ≤1%. Proteins were upregulated by 1.5-fold as compared to control. These data are also presented in Figure 3A and 3C.
