# Supplementary material for: A Multi-Mineral Intervention to Modulate Colonic Mucosal Protein Profile: Results from a 90-Day Trial in Human Subjects
Source: Nutrients. 2021 Mar 14;13(3):939. doi: 10.3390/nu13030939 (PMC8002192; doi:10.3390/nu13030939)
Supplement: Supplementary file 1 [file nutrients-13-00939-s001.zip › Table S4_Nutrients-1121446.pdf]

Table S4. Unbiased approach: Downregulated moieties.
(at 1.5-fold and <1%FDR)

Protein (Gene)	Placebo	Calcium	Aquamin	Calcium (Compared to Placebo)	Aquamin (Compared to Placebo)
Common downregulated proteins in all 3 groups (2)					
KRT6A	**0.398**	**0.302**	**0.606**	0.757	1.520
KRT17	**0.228**	**0.149**	**0.220**	0.653	0.967
Common downregulated proteins between Placebo and Calcium groups (6)					
ESRRA	**0.366**	**0.462**	2.632	1.262	7.195
KRT6B	**0.630**	**0.657**	0.785	1.042	1.246
SERF2	**0.524**	**0.626**	2.340	1.194	4.463
CSTA	**0.374**	**0.254**	0.920	0.679	2.457
ATXN3	**0.491**	**0.465**	2.104	0.949	4.288
KRT10	**0.467**	**0.665**	0.758	1.424	1.622
Common downregulated proteins between Calcium and Aquamin groups (6)					
CD99	0.824	**0.429**	**0.604**	0.521	0.733
HLA-B α49	1.823	**0.584**	**0.265**	0.320	0.145
HACD2	0.853	**0.669**	**0.548**	0.785	0.642
HLA-H	1.300	**0.315**	**0.571**	0.242	0.439
HBG2	1.021	**0.473**	**0.372**	0.463	0.365
HLA-B α59	1.946	**0.070**	**0.044**	0.036	0.023
Common downregulated proteins between Placebo and Aquamin groups (4)					
FBN1	**0.515**	0.734	**0.655**	1.426	1.272
ZNF280D	**0.557**	1.182	**0.611**	2.122	1.098
KRT2	**0.524**	1.116	**0.549**	2.131	1.048
CEP170B	**0.618**	0.844	**0.612**	1.366	0.990
Downregulated proteins - Unique to Aquamin (58)					
ZNF33B	1.177	1.344	**0.172**	1.142	0.146
UTP18	1.248	1.323	**0.184**	1.060	0.148
SUPT4H1	1.665	1.421	**0.233**	0.853	0.140
VAMP3	1.167	1.440	**0.412**	1.233	0.353
CRYZL1	1.119	1.244	**0.412**	1.112	0.368
HLA-C	1.364	0.813	**0.421**	0.596	0.309
RPL36	1.254	0.954	**0.423**	0.761	0.338
SPG21	0.998	0.705	**0.459**	0.706	0.460
FAU	1.347	1.038	**0.482**	0.771	0.358
PNLIPRP2	1.016	0.944	**0.483**	0.929	0.476
SNX17	1.195	1.134	**0.492**	0.949	0.412
PRKACG	1.189	1.354	**0.500**	1.139	0.421
CCL15	1.479	1.323	**0.510**	0.895	0.345
AMY2A	1.172	1.872	**0.517**	1.598	0.441
BLOC1S1	1.232	1.237	**0.519**	1.004	0.421
TPCN1	1.104	0.953	**0.531**	0.863	0.481
VMP1	1.270	0.985	**0.539**	0.776	0.425
AASS	1.488	1.128	**0.540**	0.758	0.363
DAZAP1	0.992	0.981	**0.542**	0.989	0.547
HIST2H3A	1.057	1.021	**0.544**	0.966	0.514
EMC3	1.200	1.164	**0.546**	0.970	0.455
RNF2	1.133	1.021	**0.557**	0.901	0.491

OSTC	1.175	0.845	**0.558**	0.719	0.475
TMEM263	1.064	1.312	**0.567**	1.233	0.532
CHTOP	1.049	1.086	**0.587**	1.035	0.560
GCG	0.964	0.906	**0.591**	0.939	0.613
ATP5J2	1.180	0.990	**0.595**	0.838	0.504
NUP43	1.063	1.048	**0.597**	0.986	0.562
RBBP9	1.224	1.136	**0.599**	0.928	0.490
PCM1	0.788	0.988	**0.613**	1.254	0.778
HLA-A	0.979	1.233	**0.620**	1.259	0.633
IGLV2-14	1.250	1.262	**0.623**	1.010	0.498
KRR1	1.212	1.079	**0.627**	0.891	0.518
DHRS13	0.865	0.876	**0.628**	1.013	0.726
POLR2F	1.254	1.200	**0.637**	0.957	0.508
CDX1	0.702	0.671	**0.638**	0.955	0.908
CAPN13	0.825	0.748	**0.638**	0.907	0.773
TANK	1.012	1.059	**0.639**	1.046	0.631
STK11IP	1.240	1.229	**0.648**	0.991	0.522
SRCAP	1.001	0.842	**0.648**	0.842	0.647
MISP3	1.143	0.930	**0.648**	0.813	0.567
FYTTD1	1.033	1.007	**0.648**	0.974	0.627
DDX3Y	0.965	0.740	**0.649**	0.767	0.672
ATF1	0.997	1.110	**0.650**	1.113	0.652
LMBRD2	1.059	1.149	**0.651**	1.085	0.615
ACOT1	1.203	0.994	**0.654**	0.826	0.544
CREBBP	0.802	0.916	**0.658**	1.142	0.820
RRP1B	0.881	1.281	**0.660**	1.454	0.749
PLEKHA1	1.224	1.093	**0.661**	0.893	0.541
TOMM5	1.076	1.048	**0.662**	0.973	0.615
IHH	1.210	1.166	**0.662**	0.963	0.547
CDX2	0.903	1.072	**0.664**	1.187	0.736
HLA-DPA1	1.319	1.241	**0.664**	0.941	0.504
SIRT3	1.176	1.222	**0.664**	1.040	0.565
RPL36AL	1.234	0.952	**0.665**	0.771	0.539
AKR1C1	1.131	1.034	**0.668**	0.914	0.590
CCNYL1	1.074	1.065	**0.668**	0.992	0.622
RPLP1	1.177	1.058	**0.668**	0.899	0.568
Downregulated proteins - Unique to Calcium (36)					
CASP14	0.684	**0.308**	1.087	0.450	1.590
ARF3	1.103	**0.319**	0.839	0.289	0.760
UAP1L1	0.888	**0.367**	0.934	0.414	1.052
CTNNA2	0.994	**0.431**	0.957	0.434	0.962
HSPA6	0.754	**0.479**	1.493	0.636	1.979
ERCC6	0.767	**0.516**	1.126	0.672	1.467
PARVG	1.062	**0.517**	0.909	0.487	0.856
TRIM38	0.850	**0.520**	0.786	0.611	0.924
NCALD	1.018	**0.528**	0.909	0.519	0.894
MT1E	1.747	**0.540**	1.492	0.309	0.854
CD19	1.202	**0.544**	1.041	0.453	0.866
NAA20	0.934	**0.547**	0.877	0.586	0.939
NACA2	0.937	**0.556**	0.973	0.593	1.039
GON7	0.839	**0.568**	0.776	0.677	0.925

MPZ	1.096	**0.573**	0.768	0.523	0.701
RANBP6	0.959	**0.575**	0.973	0.600	1.014
SPATA5L1	0.795	**0.579**	1.020	0.728	1.283
MT1X	1.578	**0.580**	0.675	0.368	0.427
TSG101	0.955	**0.583**	0.967	0.611	1.013
RPS4Y2	1.224	**0.586**	0.767	0.478	0.626
HBG1	0.882	**0.600**	0.842	0.681	0.955
PTBP2	0.748	**0.601**	1.148	0.803	1.534
INSL5	0.807	**0.614**	0.994	0.760	1.232
VPS72	0.926	**0.617**	0.901	0.667	0.973
DOPEY1	1.150	**0.621**	1.178	0.540	1.024
HLA-A	1.385	**0.633**	0.795	0.457	0.574
PIGU	0.972	**0.633**	0.939	0.651	0.966
PPWD1	0.970	**0.634**	0.843	0.654	0.868
C2orf72	1.055	**0.641**	0.925	0.607	0.876
ITGAX	0.935	**0.641**	1.367	0.685	1.461
PIGBOS1	0.959	**0.649**	1.233	0.676	1.285
F8A1	1.020	**0.649**	0.908	0.637	0.890
COX11	1.029	**0.657**	1.247	0.638	1.213
FADD	0.857	**0.663**	1.072	0.774	1.251
PHF23	0.709	**0.668**	0.729	0.942	1.029
TRABD	0.964	**0.668**	0.980	0.693	1.016
Downregulated proteins - Unique to Placebo (24)					
HLA-B α37	**0.202**	1.323	2.364	6.560	11.721
CELA2A	**0.442**	1.039	2.565	2.354	5.809
HSF1	**0.467**	0.884	0.842	1.891	1.802
KRT14	**0.475**	0.741	1.980	1.561	4.170
DCD	**0.481**	0.716	0.815	1.488	1.695
FAM109B	**0.497**	1.112	1.719	2.238	3.459
KRT1	**0.500**	0.694	1.355	1.387	2.710
BAIAP2	**0.527**	0.840	1.810	1.594	3.435
SLC39A11	**0.536**	1.007	1.675	1.878	3.122
SLC35A2	**0.541**	0.697	1.527	1.288	2.822
MYH10	**0.543**	0.988	1.388	1.821	2.558
KRT9	**0.555**	0.671	1.357	1.209	2.445
NKRF	**0.567**	1.093	1.110	1.930	1.960
FRG1	**0.592**	0.733	1.658	1.238	2.801
CELA3A	**0.610**	1.017	0.746	1.667	1.222
ZSWIM8	**0.614**	1.289	1.252	2.099	2.040
KRT77	**0.622**	0.882	1.401	1.419	2.254
KRT5	**0.631**	0.695	0.912	1.102	1.446
URI1	**0.641**	1.458	1.395	2.273	2.176
SLC12A7	**0.644**	0.781	1.749	1.212	2.713
C19orf53	**0.645**	0.753	0.989	1.166	1.533
JPT2	**0.660**	0.893	1.170	1.354	1.773
PACSIN3	**0.662**	0.799	1.710	1.207	2.583
FAM83F	**0.665**	0.892	1.093	1.342	1.645

The values represent fold-change of abundance ratio as compared to the control (all baseline samples and post-intervention placebo samples). For each downregulated protein, corresponding values from the other groups are shown for comparison. These samples were assessed by TMT based differential

proteomic expression by pooling the samples for each group. Protein FDR Confidence for all the proteins was ≤1%. Proteins were downregulated by 1.5-fold as compared to control. These data are also presented in Figure 3A and 3D.
