# Supplementary material for: A Multi-Mineral Intervention to Modulate Colonic Mucosal Protein Profile: Results from a 90-Day Trial in Human Subjects
Source: Nutrients. 2021 Mar 14;13(3):939. doi: 10.3390/nu13030939 (PMC8002192; doi:10.3390/nu13030939)
Supplement: Supplementary file 1 [file nutrients-13-00939-s001.zip › Table S6_Nutrients-1121446.pdf]

Table S6. Additional proteins of interest.

Protein (Gene)	Placebo	Calcium	Aquamin	Calcium (Compared to Placebo)	Aquamin (Compared to Placebo)
Apoptosis-related					
CARD16	0.74	0.80	1.53	1.07	2.06
BIRC6	0.90	0.96	1.65	1.07	1.83
IRF3	0.78	1.20	1.40	1.53	1.78
PPM1F	0.86	1.18	1.44	1.37	1.67
CYLD	0.93	1.20	1.49	1.29	1.59
CASP14	0.68	0.31	1.09	0.45	1.59
MIEN1	0.89	0.91	1.29	1.02	1.45
BAD	0.86	0.89	1.23	1.04	1.43
RTN3	0.91	0.94	1.30	1.03	1.43
PAWR	0.89	1.05	1.25	1.18	1.40
MAP3K7	0.76	0.96	1.06	1.26	1.40
MRPL41	0.90	0.93	1.24	1.03	1.38
NOL3	0.94	1.00	1.29	1.06	1.36
BID	0.95	0.89	1.27	0.94	1.33
Calcium metabolism-related					
CAPN14	0.48	0.74	1.75	1.54	3.64
PKIG	0.68	0.76	1.49	1.12	2.20
CRACR2A	0.78	0.94	1.37	1.20	1.75
PPP3CB	0.94	0.92	1.36	0.98	1.45
MCUR1	0.94	0.90	1.25	0.96	1.33
CPPED1	0.94	0.99	1.21	1.05	1.29
ATP2B4	0.95	1.06	1.19	1.12	1.25
CALCOCO2	0.95	1.30	1.13	1.37	1.20

The values represent fold-change of abundance ratio as compared to the control (all baseline samples and post-intervention placebo samples). These samples were assessed by TMT based differential proteomic expression by pooling the samples for each group. Protein FDR Confidence for all the proteins was $\leq$2%.
